# Astrocyte Specific Remodeling of Plasmalemmal Cholesterol Composition by Ketamine Indicates a New Mechanism of Antidepressant Action

**DOI:** 10.1038/s41598-019-47459-z

**Published:** 2019-07-29

**Authors:** Eva Lasič, Marjeta Lisjak, Anemari Horvat, Mićo Božić, Aleksandra Šakanović, Gregor Anderluh, Alexei Verkhratsky, Nina Vardjan, Jernej Jorgačevski, Matjaž Stenovec, Robert Zorec

**Affiliations:** 1 Laboratory of Neuroendocrinology-Molecular Cell Physiology, Institute of Pathophysiology, Faculty of Medicine, University of Ljubljana, Zaloška 4, 1000 Ljubljana, Slovenia; 2 Celica Biomedical, Tehnološki park 24, 1000 Ljubljana, Slovenia; 3 Department of Molecular Biology and Nanobiotechnology, National Institute of Chemistry, Hajdrihova 19, 1000 Ljubljana, Slovenia; 4 Faculty of Biology, Medicine and Health, The University of Manchester, Manchester, M13 9PT UK; 5 Achucarro Center for Neuroscience, IKERBASQUE, 48011 Bilbao, Spain; 6 Center for Basic and Translational Neuroscience, Faculty of Health and Medical Sciences, University of Copenhagen, Copenhagen, 2200 Denmark

**Keywords:** Astrocyte, Membrane lipids, Membrane fusion, Membrane fission

## Abstract

Ketamine is an antidepressant with rapid therapeutic onset and long-lasting effect, although the underlying mechanism(s) remain unknown. Using FRET-based nanosensors we found that ketamine increases [cAMP]_i_ in astrocytes. Membrane capacitance recordings, however, reveal fundamentally distinct mechanisms of effects of ketamine and [cAMP]_i_ on vesicular secretion: a rise in [cAMP]_i_ facilitated, whereas ketamine inhibited exocytosis. By directly monitoring cholesterol-rich membrane domains with a fluorescently tagged cholesterol-specific membrane binding domain (D4) of toxin perfringolysin O, we demonstrated that ketamine induced cholesterol redistribution in the plasmalemma in astrocytes, but neither in fibroblasts nor in PC 12 cells. This novel mechanism posits that ketamine affects density and distribution of cholesterol in the astrocytic plasmalemma, consequently modulating a host of processes that may contribute to ketamine’s rapid antidepressant action.

## Introduction

Major depressive disorder (MDD) affects up to 20% of the population and is the leading cause of disability worldwide^1^; therapeutic containment is frequently unsuccessful. Most current antidepressants target the monoaminergic system, which reflects the research focus on monoamines in recent decades. However, this direction is not fully justified given the widely acknowledged discrepancy between rapid modulation of monoaminergic targets and long-lasting delay of therapeutically relevant responses^2^.

Ketamine, an anesthetic and psychotomimetic, has gained much attention due to its rapid and long-lasting antidepressant effects^3^. Ketamine is considered as a non-competitive *N*-methyl-d-aspartate receptor (NMDAR) antagonist; its antidepressant action is in agreement with the glutamatergic hypothesis of MDD^4^. It postulates that NMDAR antagonism increases the synthesis of brain-derived neurotrophic factor (BDNF)^5^, which results in an antidepressant effect. Unlike classic antidepressants, a single administration of ketamine exerts fast and long-lasting effects, indicating a fundamentally distinct mechanism^6^. Ketamine treatment has a singularly wide impact on nervous tissue; it increases synaptogenesis, elevates the density of dendritic spines, and boosts the expression of α-amino-3-hydroxy-5-methyl-4-isoxazolepropionic acid (AMPA) receptors^5,7^. Furthermore, other NMDAR antagonists, in contrast to ketamine, do not produce antidepressant effects^8,9^, indicating that ketamine may affect targets other than NMDAR. Of note, the antidepressant effects of (R)-ketamine are more potent over a longer period compared to (S)-ketamine^10^, indicating specific ketamine enantiomer-mediated antidepressant activity.

The complexity of ketamine-mediated antidepressant effects has been further highlighted by a finding that ketamine modulates cyclic adenosine monophosphate (cAMP) signaling in the absence of NMDAR^11^. Signaling through cAMP is reduced in patients with depression, whereas treatment with selective serotonin reuptake inhibitors (SSRIs) increases cAMP levels^12^. Thus, ketamine may act similarly to SSRIs, since it potentiates adrenergic receptor-mediated increases in cAMP in C6 glioma cells^11^.

Ketamine also modifies presynaptic exo-/endocytotic machinery by altering the protein levels of SNARE (soluble NSF-attachment protein receptor) complex, as well as expression of vesicle-fusing ATPase, synaptotagmin-1, syntaxin-1A, synapsin-1, and syndapin-1^13–15^. Ketamine suppresses Ca^2+^ transients in astrocytes^16^, and aberrant astrocytic Ca^2+^ homeostasis may have an impact on vesicle dynamics and astroglial secretion^17^. Our previous experiments demonstrated that ketamine modulates the interaction between the vesicle and the plasmalemma by stabilizing the fusion pore in a narrow configuration^18^. This effect is reminiscent of the action of cAMP in secretory pituitary cells where cAMP promotes fusion pore flickering^19^. Hence, ketamine may act on exocytosis by affecting the cAMP signaling cascade.

cAMP may regulate synaptic activity by increasing secretion of BDNF and catecholamines^20^; in addition cAMP stimulates cellular differentiation and increases regulated exocytosis in astrocytes^21^. In other secretory cells, cAMP regulates exocytosis^22^ by acting directly on the secretory machinery^23^, increasing vesicle docking efficiency^24^ or the sensitivity of secretory proteins to Ca^2+^ ^23^, enhancing vesicle mobility^25^, or promoting homotypic intracellular vesicle-to-vesicle fusion^26^.

We tested the hypothesis that ketamine modifies astroglial secretion through cAMP signaling and confirmed that ketamine does stimulate cAMP production in astrocytes. Nonetheless, we also found that ketamine does not modulate exocytosis through cAMP, but via different mechanism(s). Ketamine is an amphipathic molecule capable of direct interaction with the plasmalemma^27^. Hence, we hypothesized that the ketamine-mediated increase in cAMP signaling may involve structural membrane changes, leading to the translocation of G-proteins in lipid rafts^11^. By visualizing cholesterol-rich plasmalemmal domains, we demonstrated that ketamine redistributes these domains in the plasmalemma of astrocytes, but not in PC12 cells and fibroblasts. This novel mechanism, capable of modulating multiple cellular functions, may well contribute to ketamine’s powerful antidepressant effect.

## Results

### Ketamine Increases [cAMP]_i_ in Astrocytes

**Figure 1:**
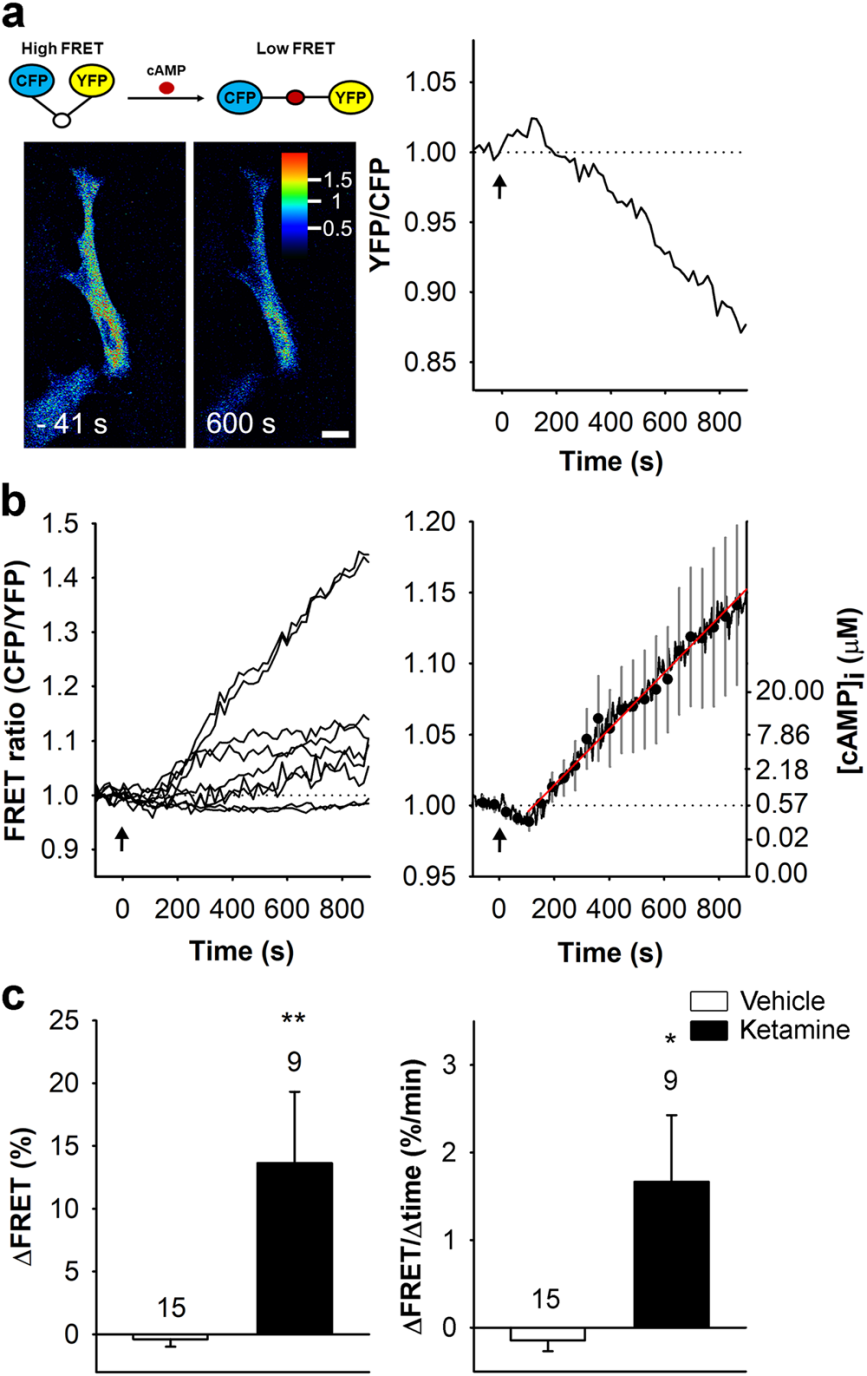
Ketamine increases [cAMP]_i_ in cultured rat astrocytes. (**a**) Schematic representation of the FRET-based Epac1-camps nanosensor activity (top) and pseudocolour FRET signal images of an astrocyte expressing the FRET-based nanosensor Epac1-camps before (−41 s) and after (+600 s) exposure to 25 µM ketamine (bottom) and the corresponding normalized time course of the Epac1-camps FRET signal (YFP/CFP). The pseudocolour scale depicts the YFP/CFP ratio values. Scale bar, 20 µm. **(b)** Time courses (left panel) and mean time course (±SE; right panel) of the Epac1-camps FRET signal in cells stimulated with ketamine (*n* = 9) at *t* = 0 s (black arrows). Data are expressed as the inverse FRET signal (CFP/YFP). The increase in the FRET signal after the addition of ketamine reflects an increase in [cAMP]_i_. The initial rate of change in [cAMP]_i_ (red line; right panel) was determined by fitting the regression line to the initial FRET signal increase (*k* = 2.2 ± 0.6%/min). Ordinate on the right shows the values of [cAMP]_i_ estimated from equation: [cAMP]_i_ = EC_50_ × ((R − R_min_)/(R_max_ − R))^1/n^ ^73^. **(c)** Mean amplitude (±SE; ΔFRET; left panel) and initial rate of the FRET signal change (ΔFRET/Δtime; right panel) in controls (white bars) and in cells stimulated with ketamine (black bars). Changes in the FRET signal are expressed as percentages of the initial values. Numbers above the error bars depict the number of cells analyzed. Mann-Whitney U test: *P < 0.05, **P < 0.01.

We monitored the effects of ketamine on the dynamics of [cAMP]_i_ in astrocytes expressing the cAMP FRET nanosensor Epac1-camps^28^ (Fig. 1a). Application of 25 μM ketamine elicited a significant (>20 µM) increase in [cAMP]_i_ that persisted throughout the recording time (900 s; Fig. 1b, right), with an average amplitude of the CFP/YFP (FRET) signal of 13.6 ± 5.7% (*n* = 9) and an initial rate of increase in the FRET signal indicating a rate of cAMP production, of 1.7 ± 0.8%/min (*n* = 9). The increase in the FRET signal started with a delay of 219 ± 52 s (*n* = 9) after onset of ketamine application. The amplitude and the initial rate of the FRET signal change in ketamine-stimulated cells differed from controls (13.6 ± 5.7% vs. −0.4 ± 0.6%; *P* < 0.01, *n* = 15) and 1.7 ± 0.8%/min vs. −0.1 ± 0.1%/min (*P* < 0.05, *n* = 15), respectively; Fig. 1). Ketamine thus increases astrocytic [cAMP]_i_.

### cAMP Facilitates Full Fusion of Larger Non-synaptic-like Vesicles

**Figure 2:**
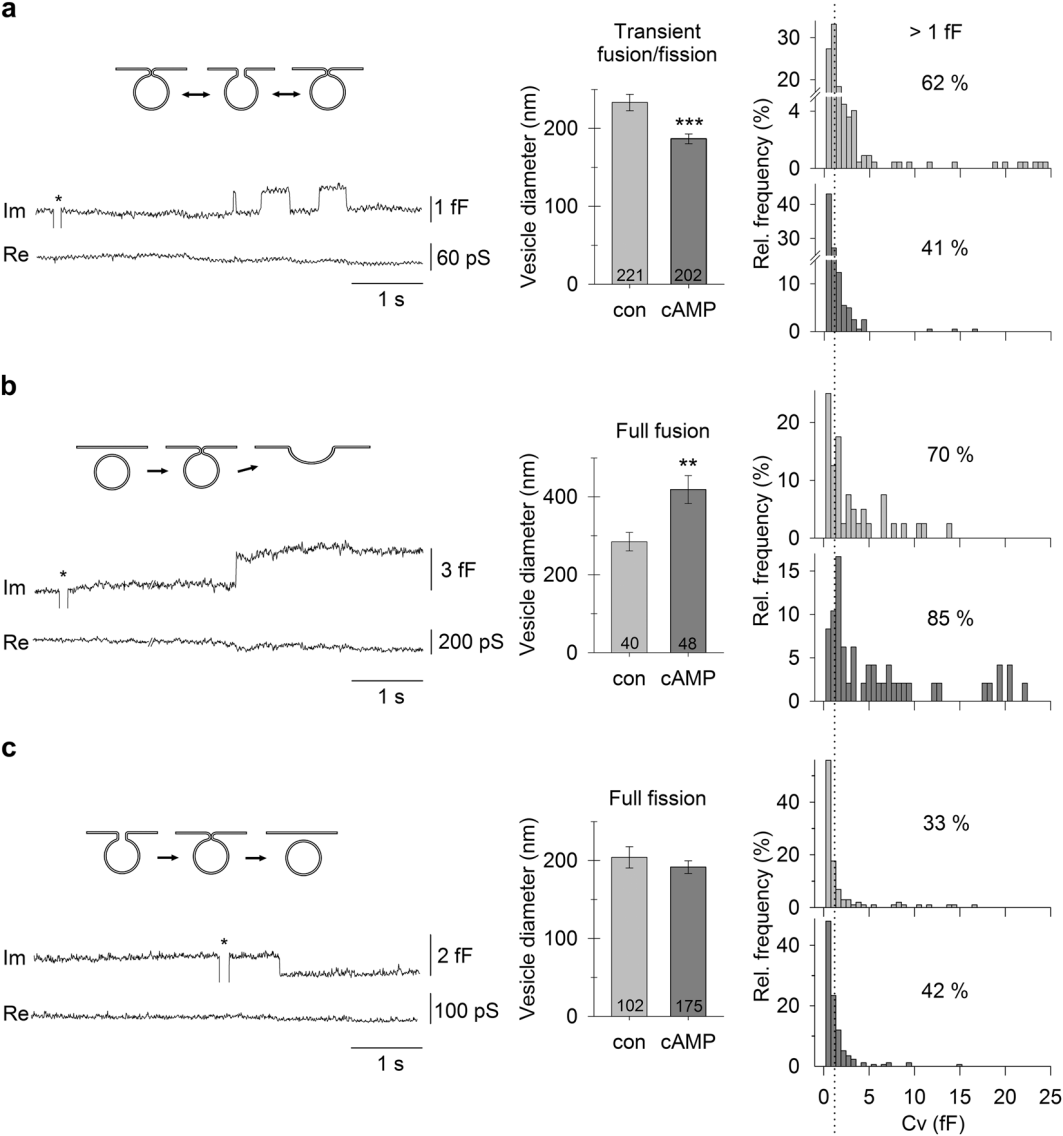
cAMP causes larger non-synaptic-like vesicles to transit to full fusion in astrocytes. **(a**–**c**) Electrophysiological recordings of the imaginary (I_m_) and real (R_e_) parts of the admittance signals (bottom) and the corresponding diagrams of vesicle interactions with the plasmalemma (top). * denote calibration pulses. (**a**) The diameter of vesicles undergoing transient fusion/fission with the plasmalemma is smaller in cAMP-treated cells (middle). Frequency distributions of C_v_ (right) reveal a greater proportion (%) of larger vesicles in untreated than in cAMP-treated cells. **(b)** The diameter of exocytotic vesicles undergoing full fusion is larger in cAMP-treated cells (middle). Frequency distributions (right) reveal a greater proportion (%) of larger vesicles undergoing full fusion in cAMP-treated cells. **(c)** The diameter of endocytotic vesicles undergoing full fission in untreated cells and in cAMP-treated cells is similar (middle), as are the frequency distributions (right). Values displayed in the graphs (%) denote the proportion of vesicles larger than 1 fF (vertical dotted line). Numbers in the bars denote vesicle number. **P < 0.01, ***P < 0.001 (Mann-Whitney U test).

Next, we examined how membrane-permeable cAMP-AM that increases [cAMP]_i_ modulates vesicle dynamics in astrocytes by measuring unitary fusion and fission membrane capacitance steps, the amplitude of which permits vesicle size estimation^29–31^. The mean diameter of vesicles that transiently fused with the plasmalemma was smaller (*P* < 0.001) in cAMP-treated (187 ± 6 nm, *n* = 202) than in control astrocytes (233 ± 10 nm, *n* = 221) (Fig. 2a). The proportion of transiently fusing vesicles with capacitance larger than 1 fF was accordingly smaller in cAMP-treated cells (41% [*n* = 83/202] vs. 62% [*n* = 138/221]). Conversely, the mean diameter of vesicles undergoing full fusion was significantly larger (*P* < 0.01) in cAMP-treated astrocytes (419 ± 36 nm, *n* = 48) than in controls (285 ± 24 nm, *n* = 40; Fig. 2b). Similarly, the proportion of full fusion of vesicles with capacitance >1 fF was larger in cAMP-treated cells (85% [*n* = 41/48] vs. 70% [*n* = 28/40]). Consistent with studies on endocrine cells^19^, our results indicate that astroglial vesicles that exhibit transient interactions with the plasmalemma (Fig. 2a, top right) in control conditions likely undergo full fusion after cAMP treatment (Fig. 2b, bottom right).

These findings were corroborated by microscopic measurements revealing that the size (surface area) and number of ANP-positive vesicles (Supplementary Fig. 1a, left), decreased after cAMP treatment (Supplementary Fig. 1a, right, and Supplementary Fig. 1b). After cAMP treatment, similar decreases were observed in vesicles immunopositive for VAMP2/3 (Supplementary Fig. 1c–f), and LC3-immunopositive autophagosomes^32–37^ (Supplementary Fig. 1g,h). These results suggest that cAMP stimulates exocytotic secretion of large non-synaptic vesicles, without affecting small synaptic-like secretory vesicles.

cAMP did not affect vesicles that underwent full fission from the plasmalemma. The mean diameter and the relative proportion of larger endocytotic vesicles were comparable in untreated and cAMP-treated cells (204 ± 14 nm [*n* = 102] vs. 192 ± 8 nm [*n* = 175] and 33% [*n* = 34/102] vs. 42% [*n* = 74/175]; Fig. 2c). In contrast, immunolabelling of early endosomes with the antibody against early endosomal marker EEA1 and vesicle loading with fluorescent dextrans revealed a cAMP-mediated effect on early endosomes. EEA1-positive endosomes were significantly larger after cAMP treatment (Supplementary Fig. 2a–c). Similarly, significantly larger dextran-loaded vesicles were observed after cAMP treatment when compared to controls; after 15 min (Supplementary Fig. 2d–f) and 3 h incubation, respectively (Supplementary Fig. 2g–i). These results suggest that cAMP stimulates intracellular fusion between early endosomes, similar to the cAMP-mediated intracellular vesicle-to-vesicle fusion reported in endocrine cells^26^.

### cAMP Favors an open fusion pore state of astroglial vesicles

**Figure 3:**
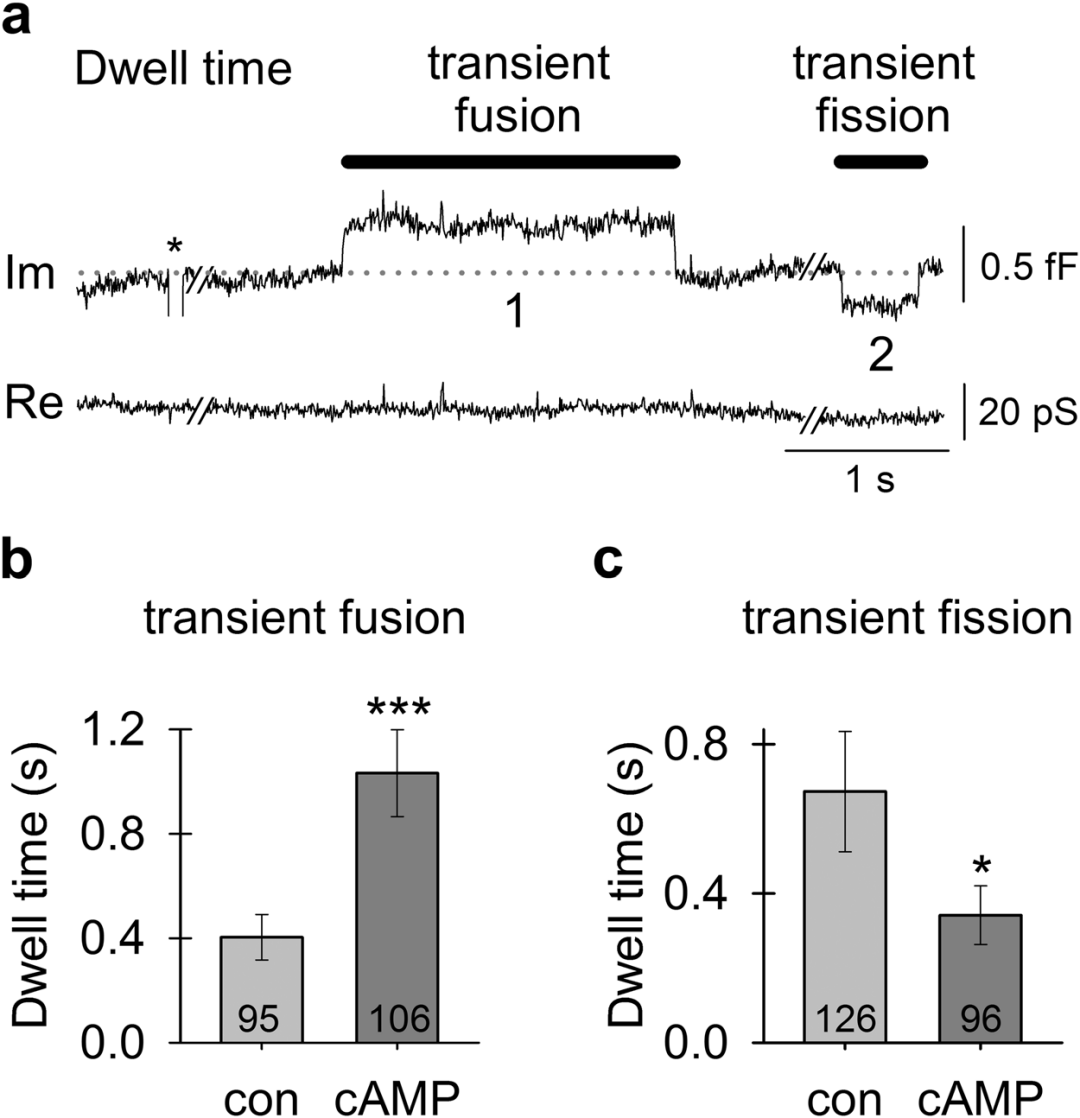
cAMP treatment favors an open fusion pore state of vesicles transiently interacting with the plasmalemma. **(a)** An electrophysiological recording of the imaginary (I_m_) and real (R_e_) parts of the admittance signal measured in a cAMP-treated astrocyte where vesicles exhibited a longer transient fusion dwell time and a shorter transient fission dwell time. The baseline is denoted by a gray dotted line. The two vesicles undergoing transient fusion and fission are denoted by the numbers “1” and “2”, respectively. * denotes a calibration pulse. **(b)** The transient fusion dwell time of an open fusion pore is increased in cAMP-treated cells. **(c)** The transient fission dwell time of a closed fusion pore is decreased in cAMP-treated cells. Numbers in the bars denote the number of transient fusions **(b)** and fissions **(c)**. *P < 0.05, ***P < 0.001 (Mann-Whitney U test).

In vesicles undergoing transient fusion/fission, the dwell time of an open or closed fusion pore can be determined (Fig. 3a); the open pore dwell time defines the time during which exocytotic cargo can be released from the vesicle lumen, whereas the closed pore dwell time defines the time of no release. Figure 3a depicts a representative recording of transient fusion and transient fission that can be interpreted as follows: at first, the baseline (dotted line) represents a state where vesicle “1” has a closed pore and vesicle “2” has an open pore. During the transient fusion, vesicle “1” opens and then closes its fusion pore to return to “baseline”. Later on in the recording, vesicle “2” closes its pore during transient fission and then reopens it to return to the “baseline”. The dwell times measured in this study ranged from 0.016 to 12.3 s. The open pore dwell time of 0.404 ± 0.087 s (*n* = 95) in controls was (*P* < 0.001) shorter than in cAMP-treated cells (1.03 ± 0.167 s, *n* = 106; Fig. 3b); this change may facilitate vesicle cargo discharge. The closed pore dwell time of 0.673 ± 0.161 s (*n* = 126) in controls was longer (*P* < 0.05) than in cAMP-treated cells (0.341 ± 0.079 s, *n* = 96; Fig. 3c). cAMP prolongs the open fusion pore configuration and shortens the closed fission pore configuration, which most likely facilitates vesicle cargo discharge. Although prolonged fusion dwell times could be related to an increase in full fusion events, our analysis indicated that cAMP had no effect on the frequency of full (or transient) fusions.

**Figure 4:**
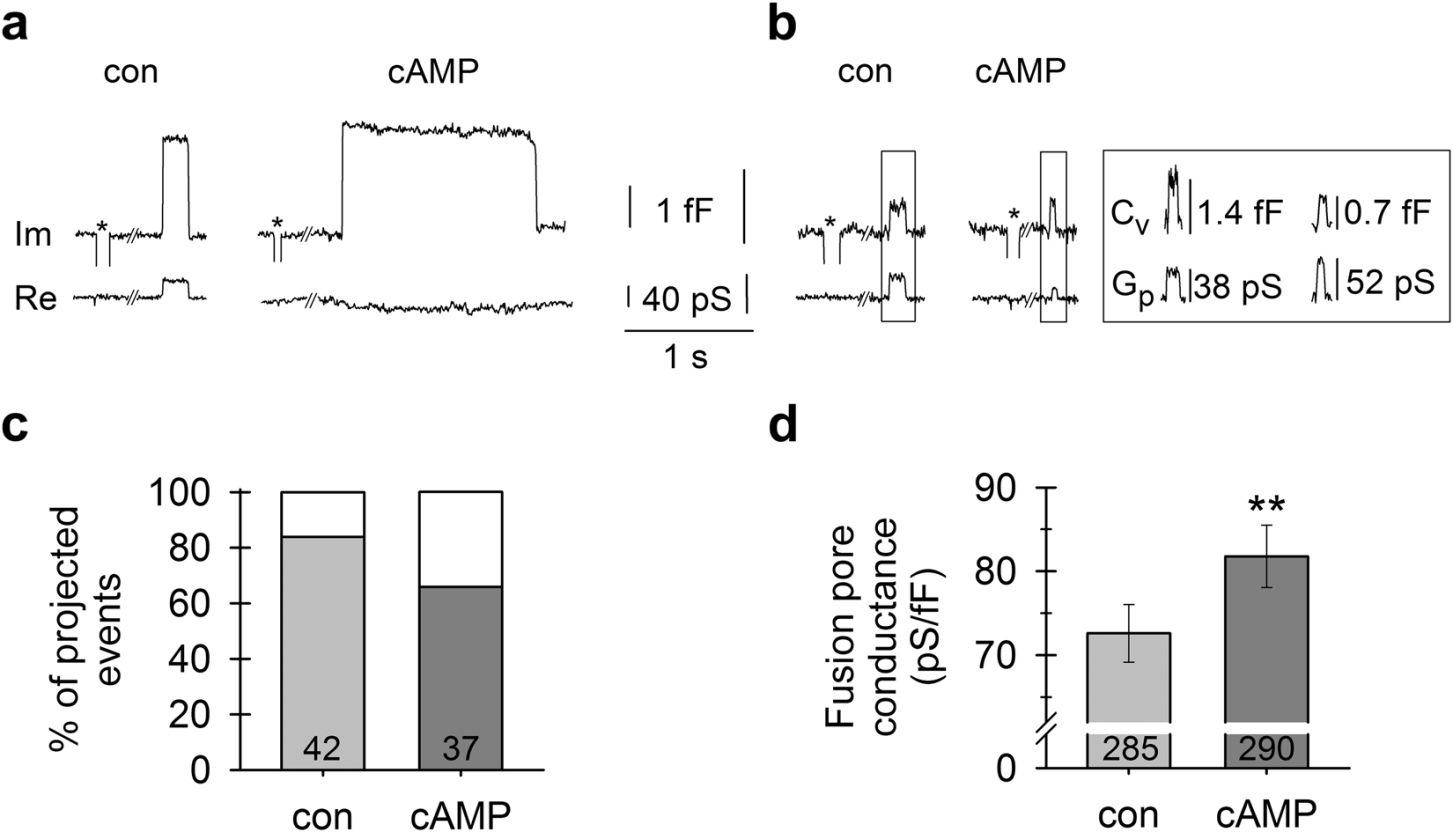
cAMP widens the vesicle fusion pore. **(a**,**b)** Electrophysiological recordings of the imaginary (I_m_) and real (R_e_) parts of the admittance signal. * denote calibration pulses. **(a)** An event in I_m_ that is projected to R_e_ in an untreated cell (left) and an unprojected event in a cAMP-treated cell (right). **(b)** A projected event with a smaller relative fusion pore conductance (G_p_) (38 pS/1.4 fF = 27 pS/fF) in an untreated cell (left) and a projected event with a larger relative G_p_ (52 pS/0.7 fF = 74 pS/fF) in a cAMP-treated cell (right). The amplitudes of the events in I_m_ and R_e_ enable calculation of vesicle capacitance (C_v_) and G_p_ (insert). **(c)** The proportion (%) of projected events. After cAMP treatment, a smaller proportion of events are projected to R_e_, indicating that a larger proportion of fusion pores are wider in cAMP-treated cells. **(d)** The relative G_p_ (pS/fF) is larger in cAMP-treated cells. Numbers in the bars denote the number of cells **(c)** and projected events **(d)**. **P < 0.01 (Mann-Whitney U test).

In contrast to ketamine^18^, cAMP increases vesicle fusion pore conductance that can be measured when discrete events in the imaginary admittance signal (*I*_*m*_) are projected to the real admittance signal (*R*_*e*_)^38^. The projection is the result of the formation of a narrow fusion pore that acts as an additional resistive element in the equivalent electrical circuit. Due to the detection limit^39^, the electrophysiological method enabled us to record fusion pores with conductance between 16.6 and 4201 pS, i.e. fusion pores with estimated diameter between 0.5 and 8.7 nm (Fig. 4a,b). Fusion pores with diameter >8.7 nm did not cause projections to *R*_*e*_ and were thus undetectable. In controls, the proportion of transient projected exocytotic events was 84% (*n* = 185/221), whereas in cAMP-treated cells, this proportion was reduced to 66% (*n* = 133/202) (Fig. 4c). Thus, the proportion of transient fusion pores with diameter >8.7 nm increased from 16% in controls to 34% in cAMP-treated cells. Correspondingly, the relative conductance of fusion pores (normalized to vesicle capacitance) was significantly larger (*P* < 0.01) in cAMP-treated cells (81.8 ± 3.7 pS/fF, *n* = 290) than in controls (72.6 ± 3.4 pS/fF, *n* = 285; Fig. 4d). These results demonstrate that cAMP widens the fusion pore, opposite to the action of ketamine^18^, which indicates that ketamine-induced vesicle fusion pore modulation is not mediated by ketamine-induced increases in [cAMP]_i_ (Fig. 1).

### Ketamine Increases the Density of Cholesterol Domains in the Astroglial Plasmalemma

**Figure 5:**
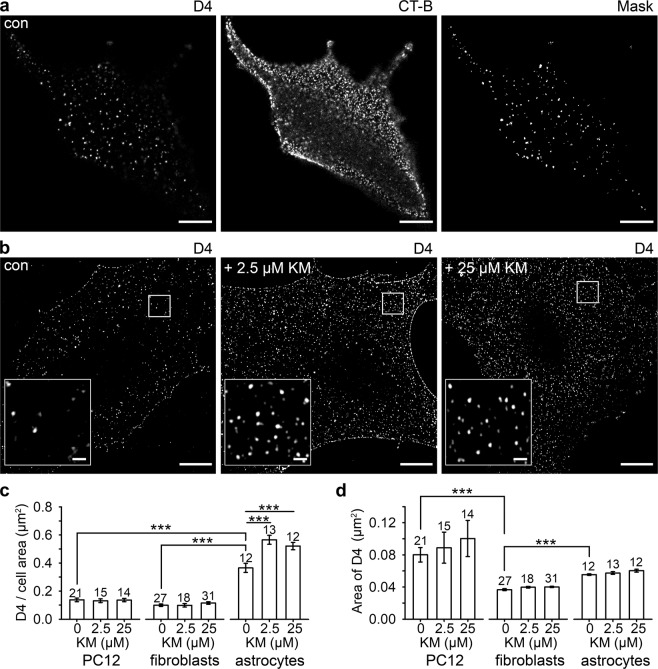
Ketamine (KM) induces an increase in the density of cholesterol-rich domains in the plasmalemma of cultured rat astrocytes. **(a)** Confocal images of cultured astrocytes double-labelled with mCherry-D4 (D4, left) that stains cholesterol-rich domains in the extracellular leaflet of the plasma membrane, and with the non-toxic B subunit toxin from *Vibrio cholerae* (CT-B, middle) conjugated to Alexa Fluor^488^, that interacts with the penta-saccharide chain of ganglioside monosialic acid (GM1), a constituent of sphingolipid- and cholesterol-enriched lipid rafts. Mask image (right) displays substantial localization of GM1 in mCherry-D4-labelled lipid rafts in astrocytes. **(b)** SIM images displaying representative mCherry-D4-labelling in non-treated astrocytes (con) and astrocytes treated with ketamine (2.5 µM KM and 25 µM KM, respectively). Insets show mCherry-D4 domains at a higher magnification. Scale bar (inset), 10 µm (1 µm). **(c)** The density of the membrane cholesterol domains (the number of cholesterol domains (D4) normalized to the cell surface area) significantly increased in astrocytes after treatment with 2.5 µM KM (0.57 ± 0.03 D4/µm^2^) and 25 µM ketamine (0.52 ± 0.03 D4/µm^2^) compared with controls (0.37 ± 0.03 D4/µm^2^) (***P < 0.001, Holm-Sidak one-way ANOVA). Ketamine did not affect the density of the cholesterol-rich domains in the PC12 cell line and fibroblasts. The density of the D4-positive domains was significantly higher in astrocytes (0.37 ± 0.03 D4/µm^2^) compared with PC12 cells (0.14 ± 0.01 D4/µm^2^) and fibroblasts (0.10 ± 0.01 D4/µm^2^) (***P < 0.001, Kruskal-Wallis test). **(d)** The average area of cholesterol-rich domains, measured in controls (treated with vehicle) and in cells treated with ketamine (2.5 µM and 25 µM) did not significantly differ in the PC12 cell line, fibroblasts and astrocytes, but it differed between different cell types. The average area of D4-positive domains was significantly higher in the PC12 cell line (0.080 ± 0.009 µm^2^) and astrocytes (0.056 ± 0.001 µm^2^) than in fibroblasts (0.037 ± 0.001 µm^2^) (***P < 0.001, Kruskal-Wallis test). The data in the graphs are reported as mean ± SEM. Numbers above the bars represent the number of cells analyzed.

As the increase in [cAMP]_i_ did not mimic the effects of ketamine on vesicle fusion/fission described previously^18^, we considered a possible direct interaction between ketamine, an amphipathic molecule, and the plasmalemma. We monitored cholesterol-rich domains in the plasmalemma of different cell types with a fluorescently tagged cholesterol-specific membrane-binding domain of perfringolysin O (D4)^40,41^. Confocal microscopy revealed a punctate D4 fluorescence pattern, representing cholesterol-rich domains to which CT-B-labelled ganglioside monosialic acid (GM1), a marker of sphingolipid- and cholesterol-rich lipid rafts, co-localized substantially (Fig. 5a); 45.6 ± 2.4% (n = 47) as determined by quantitative fluorescence co-localization analysis (CT-B vs. mCherry-D4). Super-resolution microscopy further revealed that the density of D4 domains, relative to the imaged cell area, increased after ketamine treatment in astrocytes (Fig. 5b), but not in PC12 cells and fibroblasts (Fig. 5c). In astrocytes (*P* < 0.001), the average density of D4-positive domains increased by 54% after treatment with 2.5 µM ketamine (0.57 ± 0.03 D4/µm^2^, *n* = 13) and by 41% after treatment with 25 µM ketamine (0.52 ± 0.03 D4/µm^2^, *n* = 12), compared with controls (0.37 ± 0.03 D4/µm^2^, *n* = 12). The density of D4-positive domains in astrocytes (0.37 ± 0.03 D4/µm^2^, *n* = 12) was 62% to 73% higher (*P* < 0.001) than in PC12 cells (0.14 ± 0.01 D4/µm^2^, *n* = 21) and fibroblasts (0.10 ± 0.01 D4/µm^2^, *n* = 27), respectively. Although the size of individual D4-positive domains differed (*P* < 0.001) in the cell types studied (PC12 cells, 0.080 ± 0.009 µm^2^; astrocytes, 0.056 ± 0.001 µm^2^; fibroblasts, 0.037 ± 0.001 µm^2^), these appeared unaffected by ketamine (Fig. 5d). These results indicate that ketamine alters the membrane structure selectively in astrocytes.

## Discussion

Understanding the mechanisms of the antidepressant action of ketamine is crucial for improving therapeutic strategies for MDD, a worldwide burden^1^. The clinical effects of ketamine consist of a rapid (hours) and a sustained (1–2 weeks) phases; the latter substantially outlasts the metabolic half-life of ketamine (~6 h)^3^. Although the rapid molecular pharmacology of ketamine activity is generally ascribed to NMDAR antagonism^5^, alternative pathways (such as ketamine modulation of cAMP-dependent cascades in the human brain) have been considered^12^. These pathways likely modulate brain function by primarily affecting the activity of astrocytes^42^.

Astrocytes are the primary homeostatic cells in the central nervous system that control homeostasis of major neurotransmitters, including glutamate, GABA, adenosine, and noradrenaline^43^. Major psychiatric disorders are associated with substantial decreases in astroglial density and in loss of astroglial functions, which arguably results in an imbalance in neurotransmitter homeostasis and hence in aberrant information processing in neuronal networks^44–50^. At the molecular level, mood disorders are associated with astroglia-specific changes in serotonin receptors and intracellular signaling pathways^51^. Pharmacological approaches that could specifically target astrocytes in the context of neuropsychiatric disorders have not yet been developed. Nonetheless, we present results that indicate that ketamine exerts astroglial-specific effects, which can arguably be linked to its antidepressant potential.

Ketamine, a drug with multiple targets, affects several cellular signaling cascades, including exo- and endocytosis^18,52^. Therefore, we first tested the hypothesis that ketamine regulates exo-/endocytosis through intracellular cAMP. We based this supposition on the recent finding that ketamine amplifies adrenergic receptor-mediated cAMP signaling in C6 glioma cells^11^. This in turn instigates the translocation of G_αs_-proteins from lipid rafts, allowing them to interact with and activate adenylyl cyclase^11^. It is conceivable that G_αs_-protein translocation could lead to an increase in [cAMP]_i_ even in the absence of G-protein receptor stimulation. If ketamine-induced changes in the plasmalemmal structure are mirrored as lipid raft restructuring^11^, these may activate adenylyl cyclase and increase [cAMP]_i_. Indeed, we found that ketamine increased astroglial [cAMP]_i_ (Fig. 1) in the absence of G-protein-activating neurotransmitters.

The ketamine-induced increase in [cAMP]_i_ prompted us to compare the effects of both agents on vesicle fusion. It is generally accepted that cAMP potentiates exocytotic secretion^22^ by regulating fusion of vesicles with the plasmalemma through the cAMP sensor cAMP-GEFII (Epac2)^53–55^. Protein kinases can also regulate exocytosis by increasing the population of vesicles sensitive to Ca^2+^ ^56^ with cAMP-dependent potentiation being associated with protein kinase A^22^. It was also proposed that fusion pore flickering depends on protein kinases^57^ as well as cAMP^19^. As ketamine was reported to induce fusion pore flickering^18^, this led us to question whether these effects may be mediated via cAMP.

Our in-depth analysis of vesicle fusion in astrocytes reveals a clear difference between the effects of cAMP and ketamine. Whereas cAMP increases the probability of vesicular secretion (Figs 2–4, Supplementary Fig. 1), ketamine inhibits secretion of astroglial BDNF^52^. We found no effect of cAMP on the frequency of full vesicle fusions; instead, cAMP facilitates widening of fusion pores and prolongs their open configuration (Figs 3 and 4), as was previously described in pituitary cells^19^. The ratio between fusion pore conductance and vesicle size (G_p_/C_v_) represents a measure of exocytotic secretion^58^, and cAMP increases this ratio in astrocytes. An increase in astrocyte [cAMP]_i_ promotes transient fusion of small synaptic-like vesicles (Fig. 2), as observed in pituitary cells^19^ in which Ca^2+^-dependent regulated exocytosis mediates the release of prolactin^59^. This suggests that cAMP preferentially stimulates full fusion of large non-synaptic-like vesicles^60^, whereas small vesicles remain attached to the plasmalemma with a narrow fusion pore (transient events). Consistent with this, immunocytochemical studies revealed that vesicles labelled with an antibody against VAMP2, a target of cAMP signaling and a marker of secretory vesicles^21^, are less abundant and of smaller diameter after cAMP treatment, indicating that cAMP prompts full fusion of larger secretory vesicles with the plasmalemma (Supplementary Fig. 1).

Our results also demonstrate that cAMP treatment results in the formation of larger early endosomes (Supplementary Fig. 2), suggesting homotypic vesicle-to-vesicle fusion between endosomal organelles, consistent with other studies that have suggested cAMP-evoked vesicle-to-vesicle fusion^26^. Homotypic fusion between vesicles also explains the amperometry results, which demonstrated that cAMP increased the quantal size of secretory vesicles^60–62^. We have observed a similar effect with capacitance measurements in this study, which revealed more full fusions of larger vesicles in cAMP-treated astrocytes. Our results have now demonstrated that cAMP stimulates full fusion of larger (non-synaptic-like) vesicles, widens the fusion pore and prolongs the open fusion pore dwell time, thus increasing secretory activity. Therefore, the action of cAMP contrasts with the previously reported ketamine-mediated fusion pore stabilization in a narrow state, which inhibits vesicle cargo discharge^18,52^.

This study has demonstrated that, although ketamine increases [cAMP]_i_, ketamine and cAMP have a distinct effect on vesicle fusion. Hence, we tested an alternative hypothesis whether ketamine directly alters the structure of the plasmalemma.

**Figure 6:**
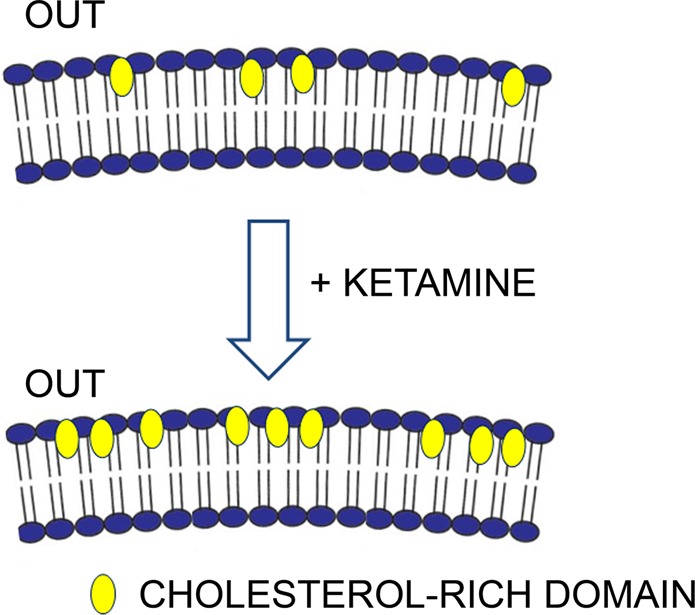
A graphical representation of the ketamine-mediated increase in density of cholesterol-rich domains in the plasmalemma of cultured rat astrocytes. Note that the fluorescent construct mCherry-D4, a specific cholesterol marker, labels cholesterol-rich domains on the outer leaflet (OUT) of the plasmalemma.

We visualized the cholesterol-rich plasmalemmal domains with a fluorescent cholesterol-specific peptide D4 from the toxin perfringolysin O^40,41^ that labels the outer leaflet of the plasmalemma. D4-positive punctate structures thus represent cholesterol-rich domains in the astrocyte plasmalemma (Fig. 5). The domain density, relative to the imaged cell area, increased significantly after ketamine treatment, yet the size of individual D4-positive domains remained the same (Fig. 5). Therefore, a relatively short exposure of cells to ketamine (30 min) results in a visible change in membrane structure. These changes are specific to astrocytes, because they were not observed in neural-like PC12 cells or in non-excitable fibroblasts (Fig. 5). Although it is unclear how ketamine specifically affects the density of cholesterol-rich domains in the plasmalemma (Fig. 6), the overall increase in cholesterol production is unlikely, since no increase in serum level of cholesterol was observed even after application of a high ketamine dose (120 and 140 mg/kg) to male Wistar rats that were sacrificed 20 min after the administration of ketamine^63^. Moreover, intraperitoneal administration of ketamine to male Wistar rats (1 mg/kg) for 6 days did not affect the cholesterol synthesis^64^. Supporting the notion that vesicle-based mechanism may be affected by ketamine through affecting the distribution of cholesterol in the plasma membrane, are experiments where the fusion of single vesicles with the plasmalemma was studied in the presence and absence of ketamine; the results revealed a robust stabilization of the fusion-pore in a narrow state, suggesting that endocytotic vesicles may not transfer from the transient fission to full fission state^18^. This may contribute to an increased density of cholesterol at the plasmalemma, since cholesterol-rich membrane domains may not be internalized via endocytosis. The availability of cholesterol appears to impair upon synapse development^65^, and our results suggest an important role of astrocytes in cholesterol homeostasis in the central nervous system. Astrocytes provide cholesterol to neurons, where it is needed for shaping neuronal structures which is particularly critical for synaptogenesis. The ketamine-induced increase in the density of cholesterol-rich domains in the astroglial plasmalemma may thus enable more intense flux of cholesterol molecules from astrocytes to neurons. We may therefore speculate that ketamine boosts plasticity in neural networks, although such a proposal requires further experimentation.

In conclusion, we here revealed that ketamine induces visible structural changes in the outer leaflet of the astroglial plasmalemma, observed as redistribution of cholesterol-rich domains. This action appears astroglia specific and may affect diverse homeostatic responses that could modulate the functional activity and plasticity of neuronal networks. In particular, these changes may influence the flux of cholesterol from astrocytes to neurons that is critical for morphological plasticity of synaptic connections^65^. In addition, structural changes of the astroglial plasmalemma likely affect adenylyl cyclase, with consequent increases in [cAMP]_i_ in the absence of G-protein-coupled receptor stimulation^11^. This new mechanism of ketamine action may explain its multiple effects on depressive behavior and highlights the role of astrocytes in the search for new antidepressants.

## Methods

### Cell Culture and Transfection

Cell cultures of primary astrocytes were prepared and enriched from the neocortex of 2–3-day-old female Wistar rats as described^52^. The care for experimental animals was in accordance with the International Guiding Principles for Biomedical Research Involving Animals developed by the Council for International Organizations of Medical Sciences and the Animal Protection Act (Official Gazette RS, no. 38/13). All experiments were performed in accordance with relevant guidelines and regulations. Specifically, we have followed the rules of Three R’s to reduce the impact of research on animals. The *ex vivo* experiments on Wistar rats were approved by the Veterinary Administration of the Republic of Slovenia (Apr. No. 3440-29/2006 and 34401-29/2009/2).

PC12 cells (ATCC CRL-1721) and fibroblasts were grown at 37 °C, in 95% air/5% CO_2_ in growth medium containing high-glucose Dulbecco’s modified Eagle’s medium (DMEM), supplemented with 10% fetal bovine serum (FBS), 5% horse serum (Gibco, Gaithersburg, MD) and 25 µg/ml penicillin/streptomycin (PC12 cells) and α-MEM, supplemented with 10% FBS, 2 mM L-glutamine and 25 µg/ml penicillin/streptomycin (fibroblasts). When cells reached the 70–80% confluence, they were plated on coverslips and used within 3 days.

The acetoxymethyl ester of cAMP (cAMP-AM, A022, BIOLOG Life Science Institute) was applied to cells in serum-free medium. To optimize cAMP-AM loading, cells were pre-incubated for 15 min in DMEM without FBS; cAMP-AM (60 µM) or DMSO (0.2%; vehicle) were applied for 30 min.

Astrocytes were transfected with the genetically encoded fluorescence resonance energy transfer (FRET)-based cAMP nanosensor Epac1-camps^28^ as described^66^. Secretory vesicles were visualized by transfecting astrocytes with pANP.emd as described^67^. The culture medium was changed after 16 h; 48–72 h after transfection, cells were incubated in serum-free medium (15 min), and with 0.2% DMSO (vehicle) or 60 µM cAMP-AM (30 min), transferred to extracellular solution (ECS) with 0.2% DMSO or 60 µM cAMP-AM for microscopic observation. Unless otherwise noted, all chemicals were from Sigma-Aldrich (Merck KGaA, Darmstadt, Germany).

### Solutions

The extracellular bath and pipette solution consisted of (in mM): 131.8 NaCl, 5 KCl, 2 CaCl_2_, 1 MgCl_2_, 10 d-glucose, and 10 HEPES/NaOH (pH 7.2). When intracellular cAMP was monitored, the bath solution consisted of (in mM) 1.8 CaCl_2_ and 2 MgCl_2_. Osmolality of the solution (300 ± 5 mOsm) was measured with a freezing-point osmometer (Osmomat030, Gonotech, Germany). cAMP-AM (60 µM) and DMSO (0.2%) were kept on ice and pipetted onto the coverslip (once a patch was established).

### FRET Measurements of Cytosolic cAMP and Data Analysis

Astrocytes expressing the Epac1-camps^28^ were examined 24–48 h after transfection with a Plan NeoFluoar 40×/1.3 NA oil differential interference contrast (DIC) immersion objective using a LSM510 META confocal microscope (Carl Zeiss, Jena, Germany). Real-time Epac1-camps FRET signal acquisition was performed as described^66^.

Unless stated otherwise, the FRET signal is reported as the ratio of the CFP/YFP fluorescence after subtracting the background fluorescence using Excel (Microsoft, Seattle, WA). The values of the FRET signals were normalized to 1.0. An increase in the FRET signal reflects an increase in the [cAMP]_i_.

Initially, astrocytes were kept in ECS and treated with 25 µM ketamine (Tocris Bioscience, Bristol, UK) for 900 s following a 100-s baseline. Control cells were treated with ECS (vehicle). The amplitude of the FRET signal (ΔFRET (%)) was determined for individual recordings by subtracting the mean FRET signal of the signal spanning the last 100 s before treatment from the first 100 s of the recording (baseline). The initial rate of the FRET signal change (ΔFRET/Δtime) was calculated for each recording as the slope of the linear regression function (ΔFRET (%) = slope (%/min) × Δtime (min)) fitting the initial FRET signal change.

### Electrophysiology

Astrocyte-coated coverslips maintained in ECS were mounted on an inverted microscope (Zeiss Axio Observer.A1). Compensated cell-attached patch-clamp recordings were performed to measure discrete step-like changes in membrane capacitance (C_m_)^68^ and fusion pore conductance^31^. Full vesicle fusion/fission was defined as a discrete upward/downward step in imaginary (*I*_*m*_) part of the admittance signal^30^ that was not followed by a step of similar amplitude (±15%) and opposite direction within 15 s, whereas transient vesicle fusion/fission was defined as a step in *I*_*m*_ that was followed within 15 s, as reported previously^18^. As C_m_ is proportional to the membrane area, the vesicle surface area and diameter (d) were determined assuming spherical vesicle geometry and a specific membrane capacitance of 10 fF/μm^2^ ^31^.

### Immunocytochemistry

Immunocytochemical staining of astrocytes treated with 0.2% DMSO or 60 µM cAMP-AM (30 min) was performed as described^52^. The following primary antibodies were used: mouse monoclonal anti-EEA1 (1:100, 610456, BD Laboratories), mouse monoclonal anti-LC3 (1:100, M152-3, MBL), mouse monoclonal anti-VAMP2 (1:2000, 104211, Synaptic Systems), and rabbit polyclonal anti-VAMP3 (1:1000, ab5789, Abcam).

### Dextran labelling

Astrocytes were incubated with 10 µM dextrans Alexa Fluor^488^ 10,000 MW (D22910, Thermo Fisher Scientific) for 15 min or 3 h and subsequently in serum-free medium (15 min) containing 0.2% DMSO or 60 µM cAMP-AM (30 min), followed by washing in PBS and fixing with 2% formaldehyde (10 min). After washing in PBS, coverslips were mounted onto glass slides using Slow Fade Gold Antifade agent (Thermo Fisher Scientific).

### mCherry-D4-PFO and EGFP-D4-PFO expression and purification

For the construction of a plasmid encoding a His-Tag-eGFP-D4 or His-Tag-mCherry-D4 fusion proteins, a DNA fragment containing eGFP or mCherry coding region was first cloned into the pGA2.1 bacterial expression vector^69^ after removing Equinatoxin II coding sequence using *XhoI* and *MluI* sites. Flexible Gly-Ser linker and additional *AvrII* restriction site were created at 3′ end of the gene encoding fluorescent protein as non-complementary ends of the amplification primers. In the next step, DNA fragment encoding for D4 domain of PFO was introduced into the prepared vector using *AvrII* and *MluI* restriction sites. The tagged D4-PFO variants were expressed in *E*. *coli* BL21(DE3) cells, which were grown in 1 l of LB medium supplemented with 100 µg/ml ampicillin (LBA) to an A_600_ of 0.5–0.7, induced with 0.5 mM isopropyl β-D-1-thiogalactopyranoside (IPTG) and incubated overnight at 20 °C, with shaking at 180 rmp. The bacteria were harvested by centrifugation for 15 min at 4 000 *g* and 4 °C, resuspended in 10 ml/g wet mass of lysis buffer (50 mM NaH_2_PO4, 300 mM NaCl, 10 mM imidazole, pH 8.0) and lysed by sonication. Cellular remnants were removed by centrifugation at 50 000 *g* for 1 hour at 4 °C. The supernatant was filtered through a 0.2-µm cellulose-acetate filter, added to the 0.5 ml 50% Ni-NTA slurry (Ni-NTA Superflow, Qiagen) and incubated at 4 °C on a rotary shaker for 60 minutes. Lysate-Ni-NTA mixture was loaded on polypropylene columns, washed twice with 10 ml wash buffer (50 mM NaH_2_PO4, 300 mM NaCl, 20 mM imidazole, pH 8.0) and the protein was eluted with 2 ml of elution buffer (50 mM NaH_2_PO4, 300 mM NaCl, 250 mM imidazole, pH 8.0). Purified protein was dialyzed (Slide-a-lyzer from Thermo Scientific, 10 000 MWCO) against 0.5 l of a buffer (50 mM Tris-HCl, 200 mM NaCl, 5% (v/v) glycerol, pH 7.4).

### Cholesterol and Ganglioside Monosialic Acid (GM1) Staining

Astrocytes, PC12 cells and fibroblasts were exposed to 2.5 or 25 µM ketamine for 30 min at 37 °C in culture medium; controls were exposed only to ECS. Cells were washed with ECS at room temperature (RT) and mCherry-D4 (0.25 µM) was applied for 30 min at RT. Then cells were washed with ECS 3× for 3 min at RT, fixed in 4% formaldehyde for 10 min at RT, and washed with ECS 3× for 3 min at RT. Samples were mounted onto glass slides with Slow Fade Gold Antifade agent. In a subset of experiments, astrocytes were first labelled by mCherry-D4 (0.25 µM) and then by the non-toxic B-subunit toxin from *Vibrio cholerae* (CT-B) conjugated to Alexa Fluor^488^ (Thermo Fisher Scientific) that interacts with the penta-saccharide chain of ganglioside monosialic acid (GM1), as described^70^.

### Structured Illumination Microscopy and Image Analysis

Astrocytes labelled with dextrans, antibodies or mCherry-D4 were imaged with a Zeiss ELYRA PS.1 super-resolution microscope with an oil-immersion plan apochromatic DIC objective (63×/NA 1.4), an EMCCD camera (andor iXon 885, Andor Technology, Belfast, UK), and 5 different grating directions for SIM.

Alexa Fluor^488^ and Alexa Fluor^546^/mCherry-D4 were excited by 488 nm argon and 561 nm DPSS laser lines, respectively, and emission fluorescence was filtered with 495–575 nm or 570–650 nm bandpass filters. The number and surface area of fluorescent vesicles were obtained by exporting tiff images to ImageJ (NIH, Bethesda, MD). To identify individual vesicles, the intensity threshold was set to 20% of the maximum fluorescence, and the minimum fluorescent spot size considered to be a vesicle was five adjacent pixels (5 × 0.04 × 0.04 µm); the minimum surface area covered by a vesicle was 0.008 µm^2^.

In mCherry-D4-stained cells, we acquired 500-nm thick z-stacked images that were analyzed in Fiji^71^. Individual cells were cropped and auto-thresholded with IsoData method to measure number and the area of D4-labelled entities in the range of 7–3000 pixels (0.01–4.80 µm^2^).

### Confocal Microscopy and Image Analysis

Z-stacked confocal images of astrocytes expressing ANP.emd were obtained with a Zeiss LSM 780 as described^17^. The number and surface area of ANP.emd-positive vesicles were obtained by ImageJ. The intensity threshold was set to 20% of the maximum fluorescence and the minimum spot size considered to be an individual vesicle was four pixels (4 × 0.088 × 0.088 μm); the minimum surface area covered by a vesicle was 0.031 µm^2^. Double-fluorescent (mCherry-D4- and CT-B-labelled) cells were observed by a plan-apochromatic oil-immersion objective 63×/NA 1.4. Z-stacked images were obtained with a 488-nm argon laser and 561-nm diode-pumped solid-state laser excitation; the emission fluorescence was bandpass filtered at 500–550 nm and 590–640 nm, respectively. Fluorescence co-localization between green-emitting Alexa Fluor^488^ and red-emitting mCherry-D4 was quantified in 8-bit TIFF files exported to ColocAna software^72^ as described^70^.

### Statistics

Data analysis was performed with SigmaPlot (Systat Software, San Jose, CA). The parameters are presented as mean ± SE. Unless stated otherwise, Student’s t test and the Mann-Whitney U test were used to determine statistical significance; *P* < 0.05 was considered significant.

## Supplementary information

Supplementary information

## Data Availability

All data generated or analyzed during this study are included in this published article and its Supplementary Information files or are available from the corresponding author on reasonable request.
