# Supplementary material for: Astrocyte Specific Remodeling of Plasmalemmal Cholesterol Composition by Ketamine Indicates a New Mechanism of Antidepressant Action
Source: Sci Rep. 2019 Jul 29;9:10957. doi: 10.1038/s41598-019-47459-z (PMC6662760; doi:10.1038/s41598-019-47459-z)
Supplement: Supplementary file 1 — Supplementary information [file 41598_2019_47459_MOESM1_ESM.doc]

**Astrocyte Specific Remodeling of Plasmalemmal Cholesterol Composition by Ketamine Indicates a New Mechanism of Antidepressant Action**

Eva Lasič, Marjeta Lisjak, Anemari Horvat, Mićo Božić, Aleksandra Šakanović, Gregor Anderluh, Alexei Verkhratsky, Nina Vardjan, Jernej Jorgačevski, Matjaž Stenovec, Robert Zorec

SUPPLEMENTARY INFORMATION

*Secretory Vesicles Are Less Abundant and Smaller After cAMP Treatment*


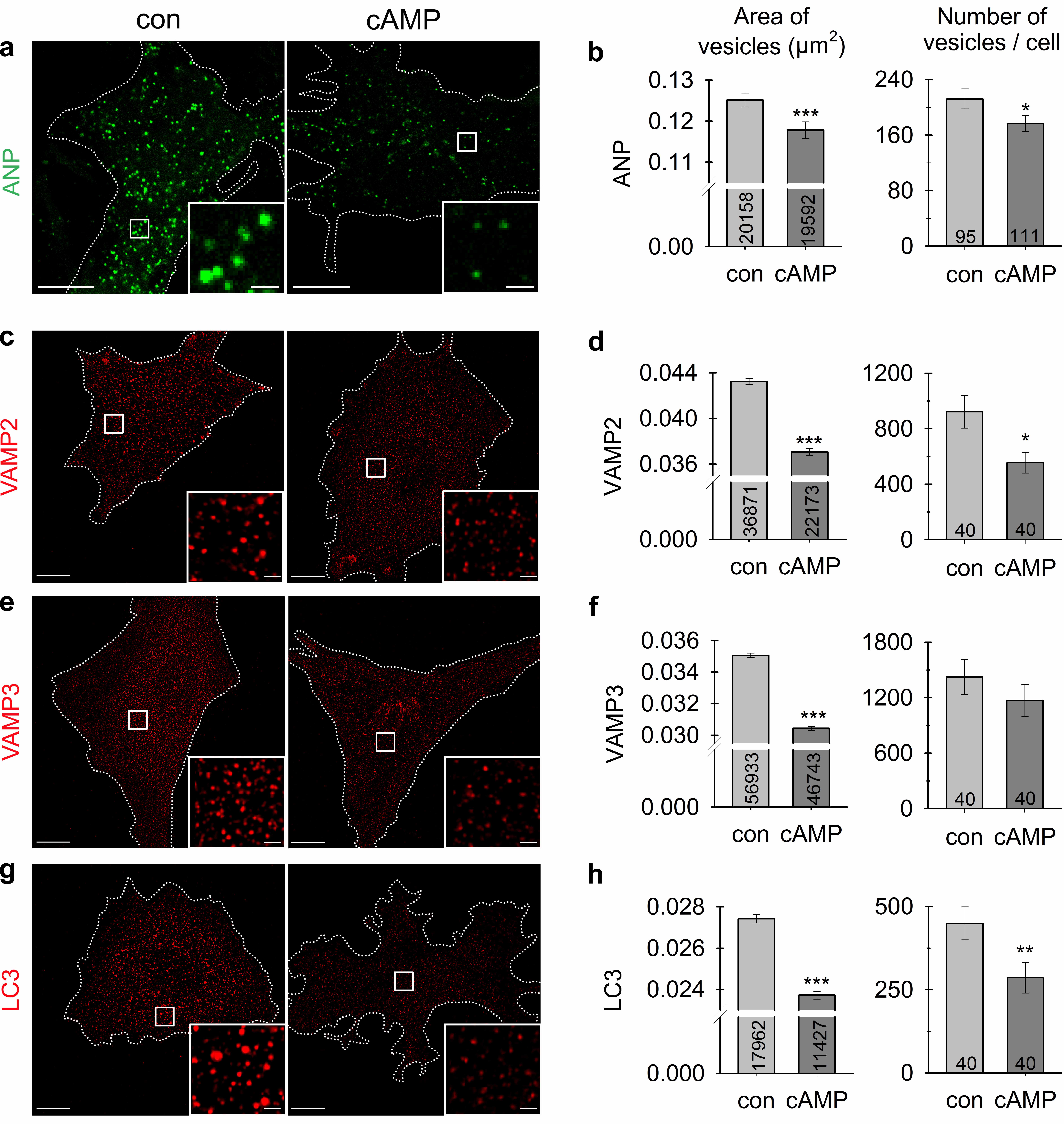


**Supplementary Fig. 1** cAMP-treated astrocytes contain fewer and smaller vesicles. Astrocytic vesicles laden with ANP.emd **(a)** and vesicles immunocytochemically labelled with antibodies against VAMP2 **(c)**, VAMP3 **(e)**, and LC3 **(g)**. Representative immunofluorescent images indicate that cAMP treatment results in fewer and smaller vesicles in astrocytes **(a,c,e,g)**. Scale bar, 10 µm, 1 µm (insets). The perimeter of individual cells is depicted by white dotted lines. In astrocytes, cAMP causes a significant decrease in the size (surface area) and number of vesicles positive for ANP **(b)**, VAMP2 **(d)**, VAMP3 **(f)**, and LC3 **(h)**. After cAMP treatment, individual astrocytes exhibit fewer vesicles positive for ANP **(b)**, VAMP2 **(d)**, VAMP3 **(f)**, and LC3 **(h)**. Numbers in the bars denote the number of vesicles (**b,d,f,h**; left) or cells (**b,d,f,h**; right). **P*<0.05, ***P*<0.01, ****P*<0.001 (Mann-Whitney U test).

## Early Endosomal Compartments Are Larger After cAMP Treatment


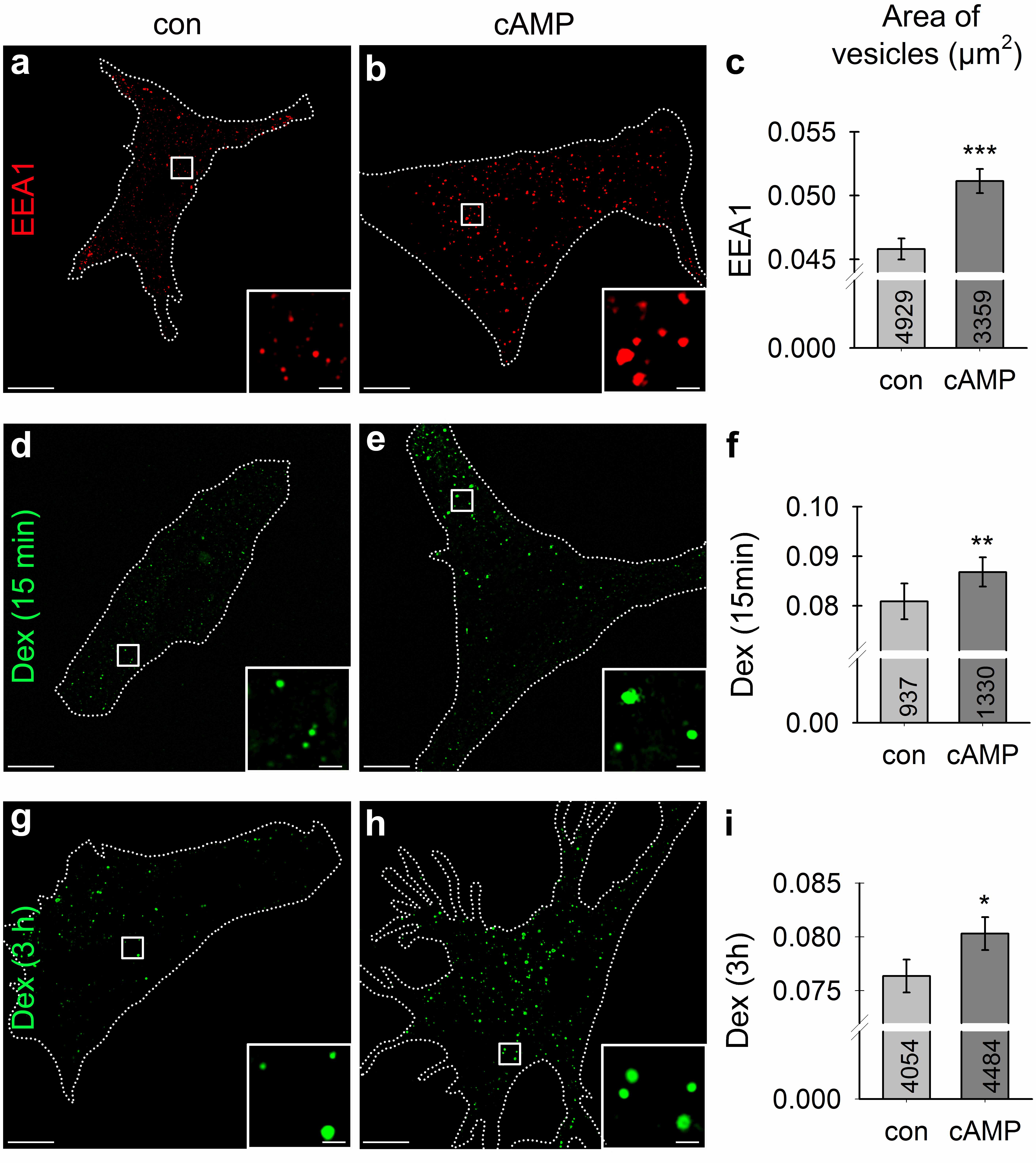


**Supplementary Fig. 2** cAMP facilitates intracellular fusion of early endosomal compartments. **(a,b)** Immunocytochemical labelling of EEA1-positive vesicles in astrocytes reveals larger vesicles in cAMP-treated astrocytes **(b)** than in untreated astrocytes **(a)**. **(c)** EEA1-positive vesicles are larger after cAMP treatment. **(d,e)** Astrocytes incubated with dextrans for 15 min reveal larger dextran-positive vesicles after cAMP treatment **(f)**. **(g,h)** Astrocytes incubated with dextrans for 3 h reveal larger dextran-positive vesicles after cAMP treatment **(i)**. Numbers in the bars denote the vesicle number **(c,f,i)**. Scale bar, 10 µm, 1 µm (insets). **P*<0.05, ***P*<0.01, ****P*<0.001 (Mann-Whitney U test).
